# Evaluating Thymic Function After Human Hematopoietic Stem Cell Transplantation in the Personalized Medicine Era

**DOI:** 10.3389/fimmu.2020.01341

**Published:** 2020-07-31

**Authors:** Ahmed Gaballa, Emmanuel Clave, Michael Uhlin, Antoine Toubert, Lucas C. M. Arruda

**Affiliations:** ^1^Department of Clinical Science, Intervention and Technology, Karolinska Institutet, Stockholm, Sweden; ^2^INSERM UMR-1160, Institut de Recherche Saint-Louis, Hôpital Saint-Louis APHP, Paris, France; ^3^Université de Paris, Paris, France; ^4^Department of Applied Physics, Science for Life Laboratory, Royal Institute of Technology, Stockholm, Sweden; ^5^Department of Clinical Immunology and Transfusion Medicine, Karolinska University Hospital, Stockholm, Sweden

**Keywords:** T-cells, thymus, hematopoietic stem cell transplantation, TREC, immune reconstitution, thymic function

## Abstract

Hematopoietic stem cell transplantation (HSCT) is an effective treatment option for several malignant and non-malignant hematological diseases. The clinical outcome of this procedure relies to a large extent on optimal recovery of adaptive immunity. In this regard, the thymus plays a central role as the primary site for *de novo* generation of functional, diverse, and immunocompetent T-lymphocytes. The thymus is exquisitely sensitive to several insults during HSCT, including conditioning drugs, corticosteroids, infections, and graft-vs.-host disease. Impaired thymic recovery has been clearly associated with increased risk of opportunistic infections and poor clinical outcomes in HSCT recipients. Therefore, better understanding of thymic function can provide valuable information for improving HSCT outcomes. Recent data have shown that, besides gender and age, a specific single-nucleotide polymorphism affects thymopoiesis and may also influence thymic output post-HSCT, suggesting that the time of precision medicine of thymic function has arrived. Here, we review the current knowledge about thymic role in HSCT and the recent work of genetic control of human thymopoiesis. We also discuss different transplant-related factors that have been associated with impaired thymic recovery and the use of T-cell receptor excision circles (TREC) to assess thymic output, including its clinical significance. Finally, we present therapeutic strategies that could boost thymic recovery post-HSCT.

## Introduction

Hematopoietic stem cell transplantation (HSCT) represent the earliest form of stem cell therapy and has been used over six decades as treatment for several malignant and non-malignant blood conditions (1). Its clinical outcome relies on a successful immune recovery, particularly an optimal T-cell reconstitution. Following HSCT, innate immune cells recover in the initial weeks to months post-HSCT, while the T-cell pool recovery is accomplished through thymic-independent homeostatic proliferation of donor-derived mature T-cells in the 1st year (2). Thereafter, the thymic-dependent *de novo* generation of naïve T-cells from donor hematopoietic stem cells (HSCs) play an essential role for immune restoring in a process that can take years and is responsible for the full restoration of TCR specificities (3). The appropriate reconstitution of the T-cell compartment is of utmost importance not only to fight opportunistic pathogens but also to promote tumor control. A prolonged post-transplant immunodeficiency is still a hurdle associated with increased infection, secondary malignancies, relapse, and high mortality rates (3). The key role played by T cells have been highlighted by the efficacy of donor lymphocyte infusions (DLI) in controlling disease relapse and by several works on T-cell reconstitution (4, 5). T-cell reconstitution, as result of thymic rebound post-transplant, is therefore strictly related to HSCT success and will be the focus of this review.

## T-Cell Reconstitution Post-HSCT

**Figure 1:**
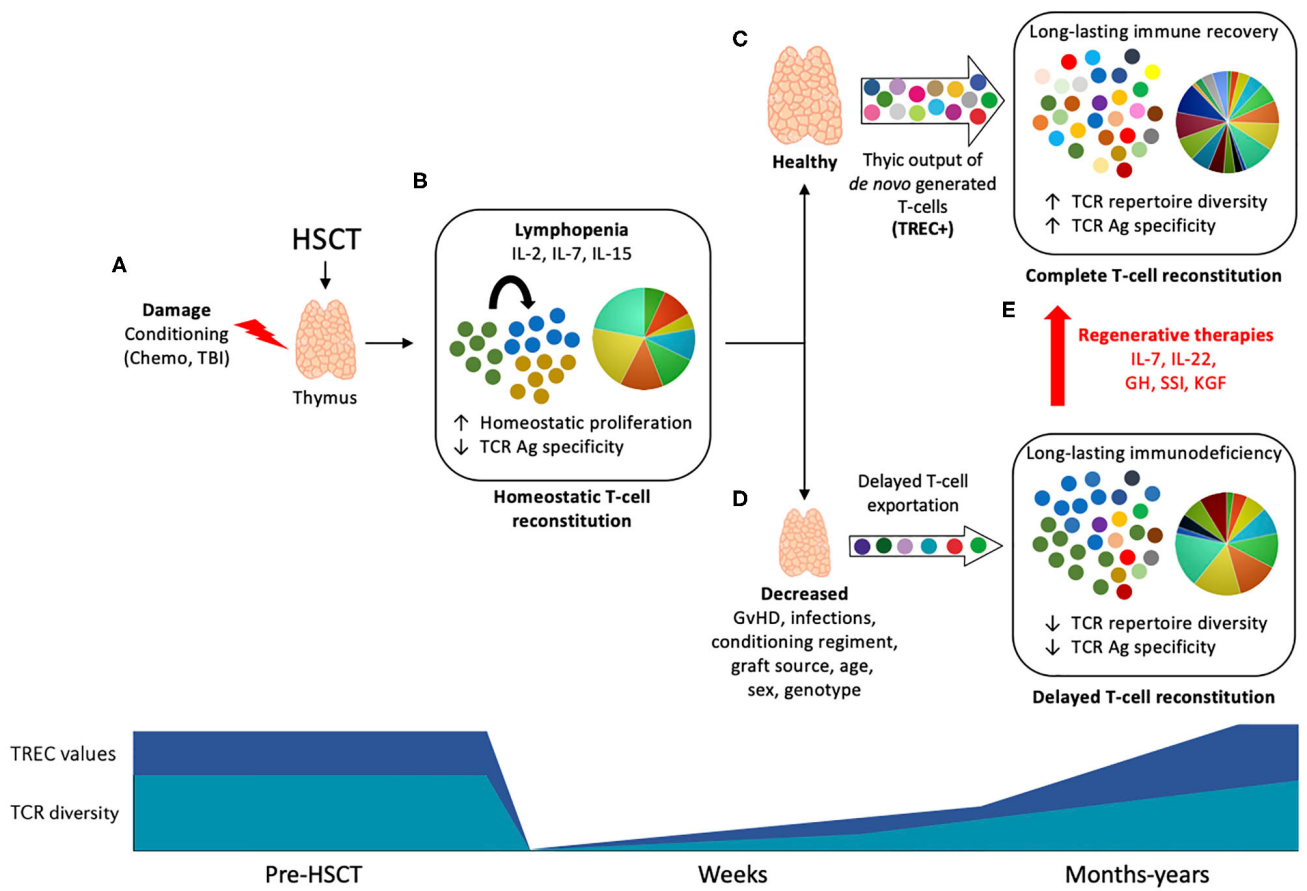
Thymic function and T-cell reconstitution post-HSCT. Upper panel: **(A)** During HSCT, the thymus is subject to damage by the conditioning drugs, corticosteroids, and other agents used in transplantation protocol, leading to impaired function. **(B)** The profound lymphopenia that follows immunosuppression leads to homeostatic expansion of residual non-depleted T cells or donor cells driven by homeostatic cytokines (IL-2, IL-7, and IL-15), resulting in oligoclonal expansion and low TCR diversity. **(C)** Within few months post-HSCT, the thymus undergoes endogenous regeneration and start to export newly generated T cells that harbor TRECs. The production of self-tolerant T cells with a broader TCR repertoire will lead to a long-lasting immune recovery and to a complete T-cell reconstitution, associated with infections control and less HSCT-associated complications. **(D)** Depending on several patient- or HSCT protocol-associated causes, the reactivation of thymic function may be limited, leading to reduced TCR diversity and impaired T-cell reconstitution, associated with increased risk of infections and high mortality. **(E)** Thymic regenerative therapies may improve thymic function post-HSCT and promote complete T-cell reconstitution. Bottom panel: TREC values (dark blue) and TCR diversity (light blue) are reduced early after HSCT and slowly increase to baseline levels in a process that can take months to years as result of thymic rebound.

The T-cell compartment recovery after transplantation occurs by two temporally and spatially distinct pathways. In the 1st weeks and months post-transplant, donor-derived, and remaining T cells not depleted by the conditioning regiment undergo peripheral expansion, comprising a thymic-independent T-cell reconstitution (Figures 1A,B) (6). This mechanism of homeostatic proliferation, also called lymphopenia-induced proliferation, is dependent of homeostatic cytokines such as IL-2, IL-7, and IL-15 (7–9). This results in the preferential expansion of CD8+ memory T cells, a subpopulation more responsive to cytokines due to previous antigen experience (such as cytomegalovirus, CMV) (10). Although thymic-independent pathway serves as a short track for rapid replenishment of the virtually empty T-cell pool, the extent to which it contributes to protect against infection is rather limited due to skewness of the TCR repertoire (11, 12). Additionally, T cells undergoing intense homeostatic proliferation are dysfunctional (13), present short telomeres (14, 15), and are more prone to activation-induced cell death (13). This altogether contributes to an incomplete T-cell reconstitution associated with high incidence of infections post-HSCT.

A complete reconstitution of the T-cell compartment depends on the thymic rebound and consequent *de novo* production of naïve T cells by the recipient thymus. Thymic-generated T cells undergo TCR rearrangement and stringent selection steps, resulting in a self-tolerant, highly diverse repertoire of polyfunctional T cells (3) (Figure 1C). This is supported by the seeding of the thymus with lymphoid progenitors arising from donor HSCs in constant maturation in the recipient's bone marrow (BM) (16). Within the thymus, T-cell progenitors undergo multi-differentiation steps and phenotypic changes, ultimately leading to the generation of a diverse repertoire of self-tolerant naïve T cells. These steps entail interactions between T-cell precursors (thymocytes), cortical thymic epithelial cells (cTECs), and stromal cells such as medullary TECs and dendritic cells (17, 18). This mechanism is tightly regulated and lead to the generation of naïve and MHC-restricted T-cells (CD4+, CD8+), with a non-self-antigen-specific TCR (19). Consequently, there is long-term immune recovery and complete T-cell reconstitution with high TCR specificities to several antigens that underlies infection control.

The thymus is a sensitive organ and suffer considerable damage during most HSCT protocols by the conditioning regimen and corticoids, and also post-transplant by infections and GvHD, leading to incomplete T-cell reconstitution and increased risk of infection and mortality (Figures 1A,D) (2). Regenerative therapies are under investigation to avoid thymic injury and to boost thymic output when appropriate (Figure 1E).

## Surrogate Markers of Thymic Output: Advances and Limitations

**Figure 2:**
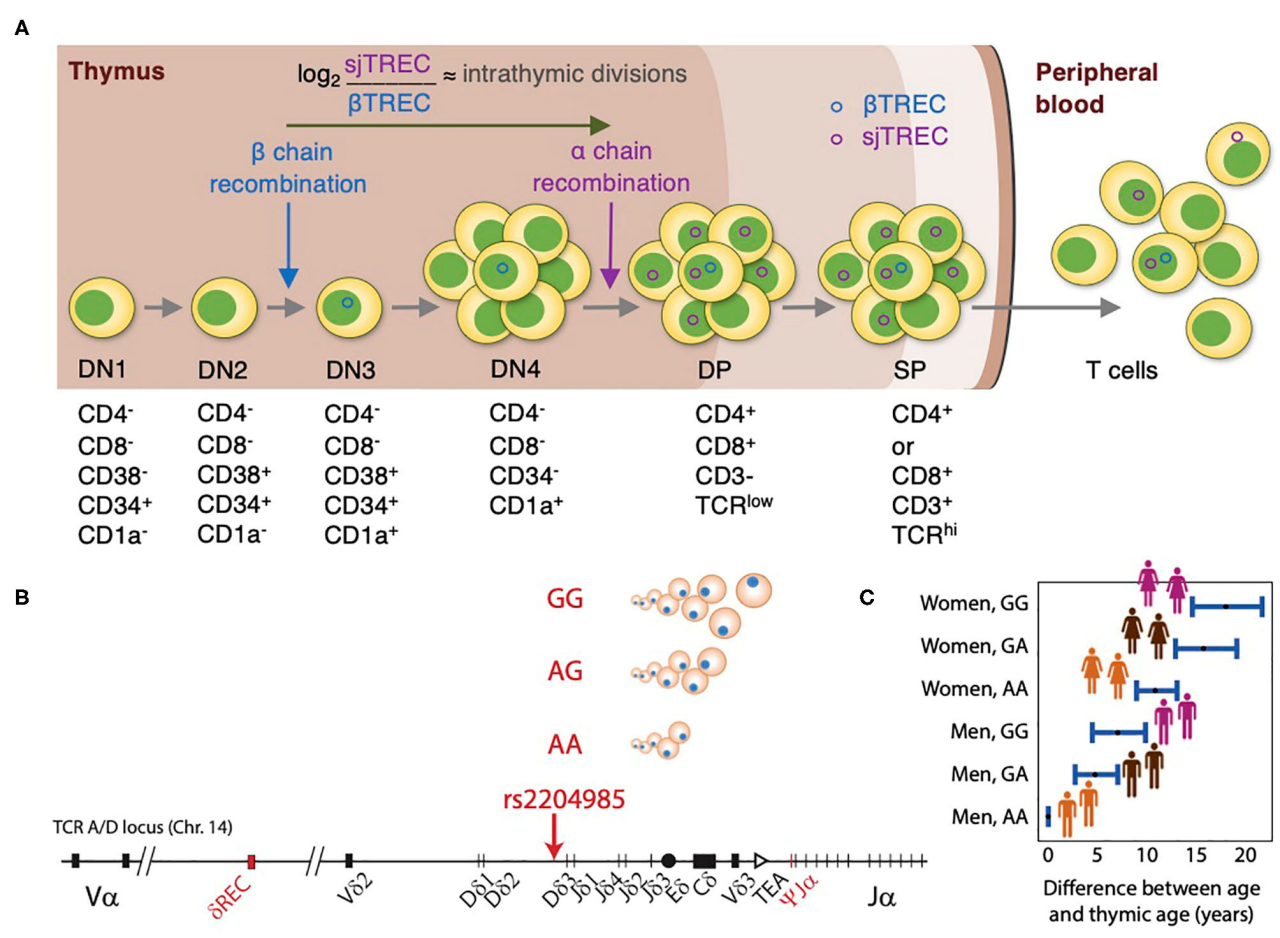
Thymic function analysis by TREC and genetic control of thymopoiesis. **(A)** In the thymus, thymocytes undergo through positive and negative selections for self/non-self-education and cell maturation. During β-chain recombination, the non-replicative episomal DNA βTREC arise from the TCRB locus excision and remain stable in the daughter cells. After differentiating and proliferating several times, thymocytes recombine the α-chain, and excise the TCRA/D locus, generating the sjTREC. The ratio between sjTREC and βTREC indicates the intrathymic proliferative activity of the thymocytes, once they remain conserved in the peripheral blood and directly reflect thymic function. **(B)** TREC values are influenced by genetic variation at the TCRA-TCRD locus, with the GG genotype at the genetic locus rs2204985 being associated with higher TREC numbers than AG and GG genotypes. **(C)** Difference between chronological age and thymic age as a function of sex and SNP rs2204985 variant. Thymic age was predicted from a regression model described in reference 26, where the age of male carrying the AA genotype are assumed as the baseline.

Understanding how the thymus contributes to T-cell reconstitution following HSCT has received considerable attention over the past years. Several efforts have been conducted to monitor thymic output, including assessment of thymic mass using computed tomography (20) and identifying recent thymic emigrants (RTE) using several phenotypic markers of naïve T cells (CD31+, CD45RA+) (21). Nevertheless, these methods either have provided semiquantitative estimates or were limited by their inability to distinguish between RTEs and long-lived naïve T cells (22, 23). TCR excision circles (TRECs) are stable episomal circular DNA fragments generated as by-products of TCR genes rearrangement and are exported from the thymus to the periphery within RTEs. Since the TCRδ locus is inserted within the TCRα locus, recombination of TCRα entails deletion of the TCRδ segment at a specific site that is common for ~70% of thymocytes, resulting in δRec-ΨJα signal joint TRECs (sjTREC) and coding joint (cjTREC) (24, 25) (Figure 2A). sjTREC values reflect the thymic output of newly generated T cells and present a strong positive correlation with naïve CD4+, CD8+, and regulatory Tcells (26). Douek et al. (25) initially introduced TRECs as a reliable surrogate marker for thymic output in the context of HIV infection.

As result of the progress in molecular techniques, besides the advancement in HSCT and the growing interest in the thymic role post-HSCT, sjTREC quantification has become feasible for many researchers and has been extensively utilized for monitoring RTE in several studies (3, 27–31). However, it is of utmost importance to take into account that in the lymphopenic setting post-HSCT, the increase or decrease in sjTREC levels does not necessarily correlate to thymic output alone (32). In fact, sjTREC levels can be influenced by other external factors such as longevity and apoptosis of naïve T cells or degradation of the sjTREC itself (33). To take into account the issue of peripheral T-cell proliferation, Ki67 staining within naïve CD4+ T-cells together with TREC quantification has been proposed to more accurately model thymic output (34).

Given the potential limitation of using sjTREC alone, a Canadian-French group developed a novel method that depends on simultaneous quantification of TRECs generated at two different thymopoietic checkpoints. βTREC is produced during DβJβ rearrangement at the DN3 stage and sjTREC generated at the DP stage (35). The estimation of βTREC provides valuable information about intra-thymic proliferation that occurs between TCRβ- and TCRα-chain rearrangements (36) (Figure 2A). Furthermore, the sj/βTREC ratio is not influenced by the dilution effect of peripheral proliferation (37). Despite the advantages of this method, it is labor intensive and time consuming which has limited its wide use. To overcome this, other researchers have developed more simplified methods for quantification of βTREC (36, 37).

## Factors Affecting Thymic Function Post-HSCT

Restoration of the normal T-cell repertoire post-HSCT is a slow and long-term process that depends on the regenerative capacity of the thymus. The over-time exportation of TREC + RTEs results in an increased TCR repertoire diversity, that does not reach baseline values until months or even years post-HSCT (Figure 1). Several parameters have been shown to influence thymic recovery following transplantation. Some of them are general, like the age, gender, and the genetic variation; while some others are transplant-specific such as conditioning, GvHD, and graft source. Here, we will briefly discuss some of these factors.

### General

#### Age

Age-related thymic atrophy/involution is a physiologic process that has been described even before revealing the immunological function of the thymus itself. Thymic function reaches its peak by the 1st year of life and gradually declines thereafter (16). It has been postulated that thymic mass decreases by an annual rate of 3% until middle age, and subsequently by 1% per year (38). In the same way, recent data have described that sjTREC values present a decrease of about 4–5% per year (26), resulting in the subsequent reduction in of naïve T-cell counts. During involution, adipose tissue gradually replaces thymic stromal cells resulting in shrinkage in its size and progressive reduction in thymopoiesis, as shown in mice (39). This leads to the involution of thymic epithelial space and reduction of number of thymocytes due the increase of thymopoiesis-suppressive cytokines (40, 41). The reduction in IL-7 (42), impaired TCRβ-chain rearrangement (43), alterations in hormones and growth factors, and changes in thymic niche have also been suggested as possible underlying mechanisms in rats and humans (44, 45). Of note, age-related involution does not lead to a complete loss of thymic function as residual thymic output can still be retained even in advanced ages, albeit significantly reduced (46). On this matter, age is an independent risk factor related to thymic function impairment in HSCT (47), with the thymic rebound post-HSCT shown to be reduced in elderly as compared to young adults and resulting in reduced naïve T-cells production (48).

Impaired immune reconstitution post-HSCT correlates with increased morbidity and mortality caused by infection and relapse (3, 49). Thus, is critical the development of strategies that enhance thymic output and immune reconstitution, particularly in elderly patients.

#### Sex

Apart from age, thymic output has also been linked to gender. Age-related thymic involution is higher in males compared to females (50) and testosterone treatment results in decreased thymic output (51). This was confirmed by two large population studies where the women presented 66–86% higher sjTREC values than men of all age ranges (26). It is therefore expected that women would have a better outcome post-HSCT with regards to infections and relapse due to the higher thymic function than men. In fact, recent data from a large cohort of around 12,000 patients have shown that recipient gender is an important prognostic factor independent of donor gender, with male recipients having inferior survival compared to females regardless of donor gender (52). This might partly be associated with the higher thymic function in females. Although a female recipient is beneficial, female donors to a male recipient has been shown to be deleterious, and a higher transplant-related mortality for male recipients of female allografts compared with other recipient-donor sex combinations was initially reported (53). Such observations led the European Group for Blood and Marrow Transplantation to include the female-to-male HSCT as a risk score, making male donors a safer choice for transplantation (54). This remark can result from the reduced thymic function in males, but it may also be due to the allogeneic response of female donor T cells toward minor histocompatibility antigens from male recipient (52).

#### Genetic Factors

Genetic background can also be implicated in determining the thymic function and the rate of thymic involution. In this regard, Clave et al. (26) have assessed the impact of 5,699,237 common single-nucleotide polymorphisms (SNPs) on sjTREC levels in a genome-wide association study of 1,000 patients from a *Milieu Intérieur* cohort. This revealed for the first time a common genetic variant (rs2204985) in a 25-kb region within the *TCRA-TCRD* locus in the intergenic Dδ2–Dδ3 segments affecting thymic output (Figure 2B). This was further replicated in an independent cohort. In both cohorts, a 43 and 44% increase of sjTREC values in GG homozygotes was observed as compared to AA homozygotes, respectively (26). The impact of this SNP was further validated in immunodeficient mice transplanted with human donor HSCs carrying the GG, GA, or AA genotype. The GG genotype was associated with a higher sjTREC and TCR diversity compared to mice transplanted with AG and AA donor genotype. Thymic age based on TREC output was then predicted from a regression model taking into account age, sex, and this genetic variation. Accordingly, the thymic function of a 40-year-old female with a GG genotype would be the same as a 21.5-year-old male with the AA genotype (Figure 2C). This study has introduced a new concept of thymic age which accounts for age, gender, and genotype (26). Their results highlight the need for personalized medicine and can be of great significance particularly in donor selection for HSCT settings. Further studies are required to reveal the clinical relevance of this SNP post-HSCT.

### Transplant-Related

#### GvHD

GvHD is a common complication post-HSCT. Although the skin, liver, gastrointestinal tract, and lung are the classical primary targeted organs, accumulating evidences also suggest the damage on the hematopoietic system (55, 56). Using sj/βTREC quantifications, Clave et al. have shown a significant reduction in thymic output in patients with acute GvHD (aGvHD). However, this effect was transient in young patients, suggesting that aGvHD-induced insults are reversible in <25 years old patients and depend on the regenerative capacity of the thymus (57). Consistent with their findings, we showed that sjTREC levels were not affected by aGvHD in a long-term follow-up study (58). Conversely, chronic GvHD was associated with decreased TREC levels regardless of disease resolution (58–60), suggesting a permanent irreversible insult. Divergent GvHD prophylaxis regimens are employed in different centers and have been suggested as possible underlying cause or long-term reduced thymic function. We then studied in a prospective randomized trial, the effect of different GvHD prophylaxis (cyclosporine/methotrexate vs. tacrolimus/sirolimus) on TREC levels post-HSCT. Results indicated no difference between the two arms of the study at any time point (60).

#### Conditioning Regimen

HSCT is preceded by cytoreductive conditioning regiments, with the aims of reducing malignant burden, avoiding graft rejection, and enhancing engraftment (61). The severity of toxicities associated with the conditioning varies according to the intensity of conditioning protocol used during HSCT. In contrast to reduced intensity conditioning (RIC), myeloablative conditioning (MAC) is associated with higher toxicity and organ damage (61, 62). Thus, it is reasonable to assume that patients receiving MAC are more prone to impaired thymopoeisis. Surprisingly, reports from several groups were inconsistent; while some studies showed rapid reconstitution in RIC recipients (63, 64), no difference or even delayed T-cell reconstitution in RIC recipients was shown by others (57, 65, 66). This controversy can be justified by realizing that RIC is mainly indicated for elderly and patients with co-morbidities. Additionally, the combination of ATG and/or DLI with RIC regimen in some works can jeopardize the real effect of mild conditioning on thymic output. Randomized trials comparing the impact of different conditioning regimens on the thymic function is warranted.

#### Graft Source

HSCs source has also been shown to impact TREC values following HSCT. However, whether peripheral blood or BM is favorable for better thymic recovery is still elusive. We have earlier shown increased TREC levels in BM recipients early post-HSCT (60, 67). Furthermore, in a recent retrospective study, we assessed TREC levels in 63 recipients after a median of 12 years post-HSCT. We found that TREC levels were higher in BM graft recipients, suggesting a beneficial role in the long-term (58). Despite other studies have not shown a significant association between TREC and stem cell graft source (59, 68), a higher engraftment and supportive thymic function is expected in BM grafts, which contain mesenchymal stromal cells and dendritic cells that can possibly engraft in the host after HSCT and support hematopoiesis.

## Clinical Significance of Monitoring Thymic Function in HSCT Setting

Monitoring thymic output in HSCT patients have significantly improved our understanding main issues related to HSCT and has allowed researchers to identify factors affecting thymic recovery post-HSCT. The association between pre-transplantation TREC values and survival established thymic function to be a reliable predictor for morbidity and mortality (3, 69). Additionally, the clearance of CMV viremia and survival after umbilical cord blood (UCB) depends on a successful reconstitution of thymopoiesis (70, 71). In this regard, the monitoring of TREC levels in 331 samples from 158 allogeneic HSCT patients showed a strong correlation between low TREC levels and opportunistic infection in the first 6 months post-HSCT (72). Moreover, low TREC levels post-HSCT has been associated with higher incidence of relapse (49). Similarly, results by Wils et al. (73) indicated a significant reduction in incidence of severe infections and lower risk of non-relapse mortality in patients who showed effective thymic recovery early post-HSCT. In children undergoing T-cell depleted haploidentical HSCT, Clave et al. (74) showed that the incidence of leukemia relapse was found to be higher in patients who had undetectable βTREC and low sjTREC levels post-HSCT. The same group has also reported similar findings in children who underwent UCB-HSCT (75). In a retrospective study, we earlier showed increased OS and decreased transplantation-related mortality in patient who had higher TREC levels at 3 months post-HSCT (67). In another study, we found high TREC levels post-HSCT to be associated with improved survival and decreased relapse incidence in leukemia patients (76). Corroborating with these results, Torlen et al. (60) reported reduced transplantation-related mortality and increased 5-year OS in patients with high TREC levels in the first 6 months post-transplantation. Of note, the thymic rebound post-transplantation is associated with a favorable clinical response to autologous HSCT in autoimmune disease patients as well (71).

## Boosting Thymic Output Post-HSCT

Despite the thymus can spontaneously restore its function post-HSCT, depending on recipient's age or repeated insults suffered during transplantation protocol, thymic regeneration might be impaired for long periods. Enhancing thymic function leading to efficient T-cell reconstitution might be a promising route for future HSCT. In this regard, several strategies have been investigated yet so far only few have been successfully translated to the clinic (Figure 1E) (2).

Growth hormone (GH) is one of the somatotropic hormones with a pivotal role in hematopoiesis. In a prospective randomized trial, Napolitano et al. investigated the impact of daily subcutaneous injections of rGH for 6 months on thymic function of HIV-infected patients. They demonstrated increased thymic output and TREC levels in treated patients (77). Corroborating with these findings, Hansen et al. (78) reported in a randomized placebo-controlled trial increase in thymic indices in HIV group treated by low dose rGH. Although GH treatment has been used in children undergoing HSCT to treat post-radiation growth disorders (79, 80), its role in immune reconstitution has not so far been well-investigated.

The observation of rapid decline of thymic function with onset of puberty has suggested a role of sex hormones in thymic involution and set sex steroid inhibition (SSI) as a feasible strategy to restore immune competence in immunodeficient individuals (81). In fact, SSI surgically or pharmacologically has been associated with improved thymic function following HSCT in humans (82, 83), indicating that this approach can be used to boost thymic regeneration.

Keratinocyte growth factor (KGF) is produced by thymocytes and other stromal cells in the thymus and act inducing the expansion of epithelial cells. Several studies showed that administration of KGF alone or in combination with androgen blockade before HSCT is associated with improved regenerative capacity of the thymus and efficient T-cell reconstitution post-transplantation (84). Additionally, it has been shown that KGF protects TECs from the radiation-, conditioning-, and GvHD-induced damage in murine models (85–87), but its effects in humans are still elusive.

In addition to the above described strategies, several studies on murine models highlighted the role of IL-7, IL-21, IL-22, and zinc in restoring normal thymopoeisis (42, 88–93). In fact, rIL-7 administration after human allogeneic T-cell depleted HSCT demonstrated an increase in T-cell recovery and increased TCR diversity, but no significant increase on thymic output was reported (94). Additionally, the advances in thymic organoids bioengineering technologies can provide a novel solution in the future (95, 96).

## Concluding Remarks

The understanding of thymic function on HSCT outcomes have evolved tremendously in the last decades. Recently, the role of recipient age and sex and donor genotyping has been unrevealed as important factors associated with HSCT response, together with GvHD, conditioning regiment, and graft source. The remarkable finding that a donor genetic SNP affects strongly the recipient thymic output open a new era of graft selection (26), especially in cases when the recipient would benefit from an improved thymic function. Additionally, the recently published thymus cell atlas paved the way for a better understanding of human T-cell development and may impact HSCT research in the near future (97). The challenge for the next decades will be to translate these advances into a better donor selection and how to identify the proper recipient that should be treated with thymic boosters and which one to be used.

## Author Contributions

AG, EC, MU, AT, and LA prepared the figures, wrote the manuscript, and revised the final version. All authors contributed to the article and approved the submitted version.

## Conflict of Interest

The authors declare that the research was conducted in the absence of any commercial or financial relationships that could be construed as a potential conflict of interest.
